# MDPNet: a dual-path parallel fusion network for multi-modal MRI glioma genotyping

**DOI:** 10.3389/fonc.2025.1574861

**Published:** 2025-05-19

**Authors:** Huaizhi Wang, Haichao Liu, Fang Du, Di Wang, Xianhao Huo, Jihui Tian, Lijuan Song

**Affiliations:** ^1^ School of Information Engineering, Ningxia University, Yinchuan, Ningxia, China; ^2^ Ningxia Key Laboratory of Artificial Intelligence and Information Security for Channeling Computing Resources from the East to the West, School of Information Engineering, Ningxia University, Yinchuan, Ningxia, China; ^3^ Collaborative Innovation Center for Ningxia Big Data and Artificial Intelligence Co-founded by Ningxia Municipality and Ministry, School of Information Engineering, Ningxia University, Yinchuan, Ningxia, China; ^4^ School of Advanced Interdisciplinary Studies, Ningxia University, Zhongwei, Ningxia, China; ^5^ General Hospital of Ningxia Medical University, Yinchuan, Ningxia, China

**Keywords:** glioma, magnetic resonance imaging, multimodal, heterogeneity, dual-path genotyping

## Abstract

**Background:**

Glioma stands as one of the most lethal brain tumors in humans, and its accurate diagnosis is critical for patient treatment and prognosis. Magnetic Resonance Imaging (MRI) has been widely utilized for glioma diagnosis and research due to its non-invasive nature and clinical accessibility. According to the 2021 World Health Organization Central Nervous System Tumor Classification guidelines, glioma subtypes can be determined through molecular status information of Isocitrate Dehydrogenase (IDH), Chromosome 1p/19q codeletion (1p/19q), and Alpha Thalassemia/Mental Retardation Syndrome X-linked (ATRX) genes.

**Method:**

In this study, we propose a dual-path parallel fusion network (MDPNet) designed to comprehensively extract heterogeneous features across different MRI modalities while simultaneously predicting the molecular status of IDH, 1p/19q, and ATRX. To mitigate the impact of data imbalance, we developed a cross-gene feature-sharing classifier and implemented an adaptive weighted loss function, substantially enhancing the model’s predictive performance.

**Results:**

In this study, each gene classification task was formulated as a binary classification problem. Experiments conducted on public datasets demonstrate that our method outperforms existing approaches in accuracy, Area Under the Curve (AUC), sensitivity, and specificity. The achieved classification accuracies for IDH, ATRX, and 1p/19q reach 86.7%, 92.0%, and 89.3%, respectively. The source code of this study can be viewed at https://github.com/whz847/MDPNet.

**Conclusion:**

The proposed framework exhibits significant advantages in integrating heterogeneous features from multi-modal MRI data. Experimental results from internal datasets further validate the model’s superior generalizability and clinical utility in assisting glioma diagnosis, highlighting its potential for real-world clinical applications.

## 1 Introduction

Gliomas are the most common primary brain tumors in the central nervous system (1). According to the 2021 World Health Organization Central Nervous System Tumor Classification (WHO CNS5), gliomas are classified as low-grade gliomas (LGG, grades 1-2) and high-grade gliomas (HGG, grades 3-4) (2). Low-grade gliomas are less invasive and have a more favorable prognosis compared to high-grade gliomas (3). Depending on the cell type, gliomas are mainly divided into glioblastoma, astrocytoma, and oligodendroglioma. In the WHO CNS5 classification, glioblastoma is explicitly classified as the most malignant high-grade glioma (grade 4), while astrocytomas and oligodendrogliomas can be classified as low-grade (grade 2) or high-grade (grades 3 to 4) based on their molecular characteristics and pathological characteristics (2). Treatment strategies and survival outcomes differ significantly between glioma subtypes, making an accurate classification crucial to determine appropriate treatment plans and evaluate prognosis.

According to the WHO CNS5, the types of gliomas can be determined based on the status information of gene molecules such as IDH, 1p/19q, and ATRX. Specifically, IDH and ATRX can be classified into two categories based on mutation status: wild-type and mutant. Meanwhile, the 1p/19q status is determined by the presence or absence of co-deletion of chromosome 1p and chromosome 19q, categorized as intact or co-deleted. If an IDH mutation is present along with a 1p/19q co-deletion, the tumor is classified as an oligodendroglioma. If there is no 1p/19q co-deletion but an ATRX mutation is present, the tumor is classified as an astrocytoma. In cases where IDH remains wild-type, the tumor is diagnosed as a glioblastoma (2). IDH is a key enzyme involved in cellular metabolism and its mutation status is crucial to the diagnosis and treatment of gliomas. In general, gliomas with IDH mutations have a more favorable prognosis than those with wild-type IDH (4, 5). The 1p/19q codeletion is a rare chromosomal loss event in gliomas (6). ATRX is a protein involved in DNA repair and replication, and its mutation status also affects the diagnosis and prognosis of gliomas (7).

**Figure 1:**
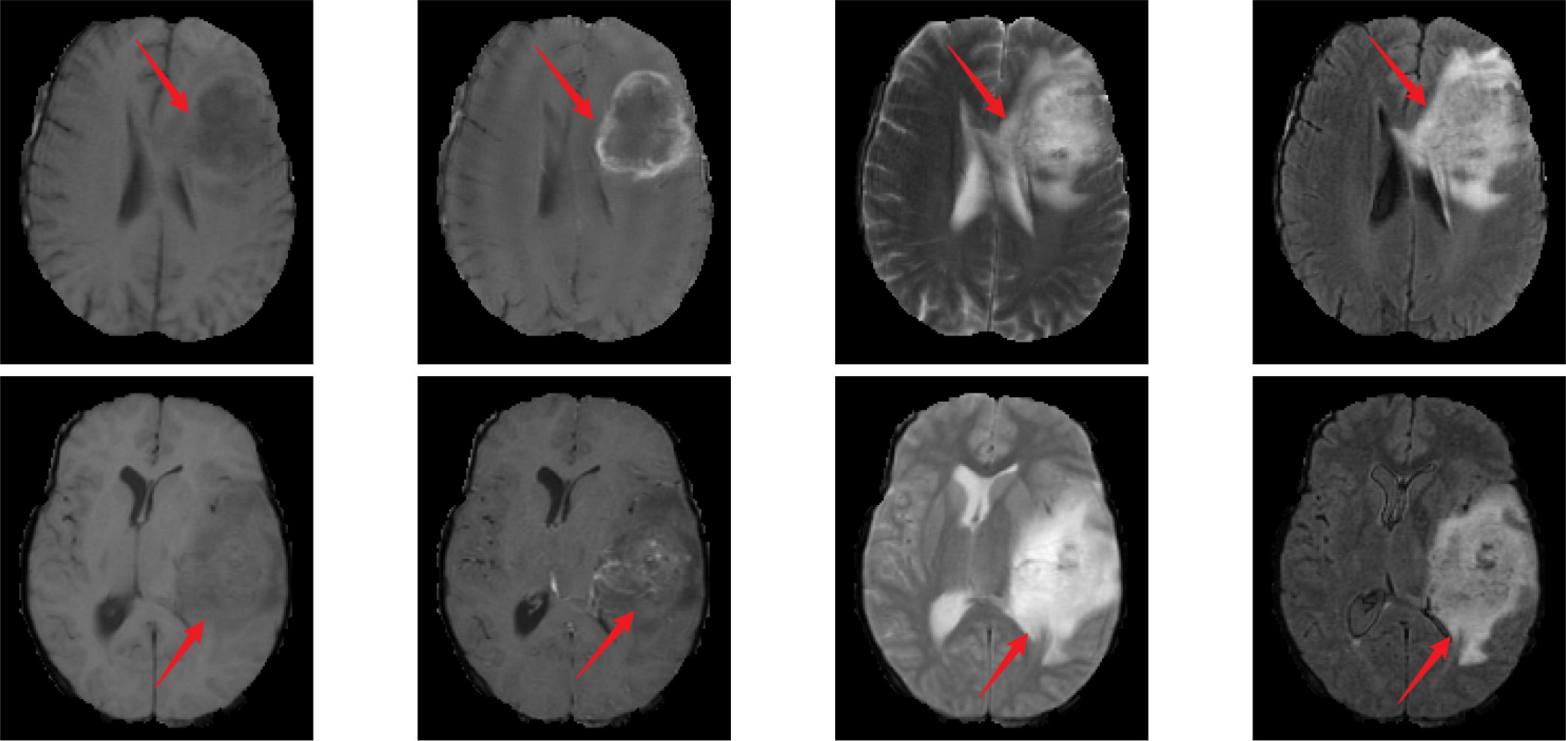
MR images of different modalities. From left to right, the images correspond to T1, T1-ce, T2, and FLAIR modalities. The first row represents cases with IDH wild-type, ATRX wild-type, and 1p/19q non-codeletion status. The second row represents cases with IDH mutant, ATRX mutant, and 1p/19q non-codeletion status.

Traditionally, the gold standards for identifying these gene statuses mainly include immunohistochemistry and gene sequencing, both of which typically rely on tissue samples obtained through surgery. Although these methods form the basis for precise diagnosis, they require postoperative procedures, which leads to delays in the obtaining of molecular diagnostic information (8, 9). In contrast, if gene status information could be obtained preoperatively through non-invasive imaging analysis, it would provide supplementary support for surgical planning and postoperative treatment strategies. Therefore, exploring non-invasive preoperative detection methods holds significant value in optimizing treatment strategies and minimizing patient burden. Magnetic resonance imaging (MRI) is widely used due to its non-invasive nature and diagnostic utility. It can acquire different sequence modalities of the patient’s brain, such as T1, T1-ce, T2, and FLAIR. These modalities provide information such as the morphology, spread range, and surrounding tissues of gliomas (10). Among them, the T2 and FLAIR modalities mainly provide information such as lesion boundaries, edema, and invasion related to glioma lesions (11). **Figure 1** presents MRI scans from the four modalities discussed.

In **Figure 1**, the MRI in the first row shows IDH wild-type, ATRX wild-type, and 1p/19q non-codeletion; the MRI in the second row shows IDH mutation, ATRX mutation, and 1p/19q non-codeletion. The red arrow in the image indicates the glioma lesion area. Normally, IDH wild-type gliomas exhibit peripheral annular enhancement with accompanying necrosis; however, most IDH-mutant gliomas demonstrate less contrast enhancement and more non-enhancing solid components (12). Compared to ATRX wild-type gliomas, ATRX-mutant gliomas exhibit a lower frequency of tumor edema (13). Gliomas with 1p/19q codeletion often have indistinct tumor margins and commonly contain calcifications (12). In clinical practice, due to the heterogeneity of gliomas, even experienced neuroradiologists often struggle to accurately differentiate glioma types and genotypes directly from MRI scans.

In recent years, deep learning algorithms have been extensively applied in radiomics studies of gliomas, demonstrating remarkable progress in molecular subtyping based on multi-modal MRI and genomic profiles (14, 15). However, existing deep learning-based approaches for glioma genotyping using multi-modal MRI predominantly focus on individual classification of IDH or 1p/19q, neglecting inter-gene correlations (16–18). This limitation not only constrains model performance but also diminishes clinical utility. Consequently, simultaneous joint classification of three molecular markers (IDH, 1p/19q, and ATRX) within a unified deep learning framework represents a promising research direction.

Gliomas are biologically and morphologically highly heterogeneous brain tumors, with this heterogeneity constituting a fundamental aspect of the disease and a critical feature that deep learning models must effectively capture (19).Previous studies have indicated that the T2-FLAIR mismatch serves as a significant biomarker for predicting gene mutations, such as IDH and 1p/19q, characterized by high heterogeneity (14). As illustrated in **Figure 1**, the characteristic heterogeneity between T2 and FLAIR is demonstrated as follows: gliomas exhibit nearly complete and uniformly high-intensity signals on T2 imaging, while FLAIR imaging reveals high-intensity peripheral edges and relatively low intensity central regions. This high feature heterogeneity in T2 and FLAIR is crucial for the prediction of genes such as IDH. As these heterogeneous features can be used to identify gliomas with mutations in such genes. Therefore, how to effectively utilize the heterogeneous features between T2 and FLAIR and adaptively fuse them with other discriminatory features related to such genes has become a key issue in improving the prediction performance of those genes.

The issue of data imbalance is highly prevalent in medical imaging data analysis, posing significant challenges for disease classification tasks (20). When a particular disease constitutes only a small fraction of the entire dataset, deep learning classifiers tend to favor the majority class, thereby underestimating the importance of the minority class. In such scenarios, although the network may achieve high disease prediction accuracy on the training and validation sets, its actual predictive performance—often evaluated using metrics such as the area under the curve (AUC)—frequently falls short. This discrepancy can lead to misdiagnoses, ultimately compromising patient outcomes. Therefore, developing effective strategies to mitigate the adverse impact of data imbalance on network models and enhance their clinical applicability remains a critical research priority.

**Figure 2:**
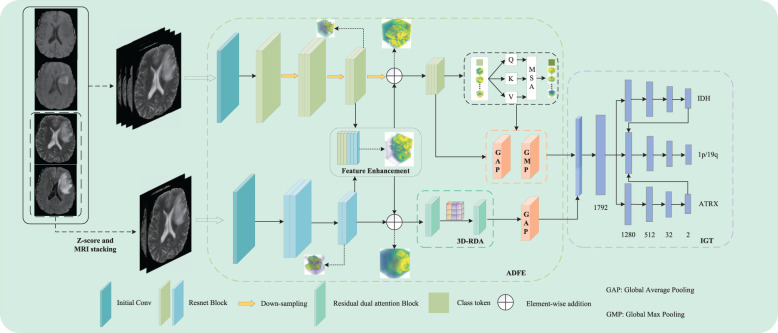
The network architecture of MDPNet.

In this study, we propose a dual-path parallel fusion network (MDPNet),as shown in **Figure 2**, which performs joint classification of gene molecules from multimodal MRI in an end-to-end manner. The network comprises two core components: 1) attention-guided asymmetric dual-path feature extraction (ADFE), integrating convolutional neural networks with attention mechanisms to comprehensively capture local global information and T2-FLAIR heterogeneous features; 2) gene typing guided by inter-gene relationships (IGT), which constructs a sophisticated classifier by modeling gene-gene interactions to achieve accurate classification of IDH, 1p/19q, and ATRX. Furthermore, a tailored loss function is introduced to mitigate the adverse effects of data imbalance during model training. These methodological advancements not only address the limitations of existing studies by enhancing both the comprehensiveness and accuracy of predictions but also provide robust support for precision medicine in glioma diagnosis and treatment.

## 2 Methodology

In this study, we propose a novel dual-path parallel fusion network model (MDPNet) for glioma genotyping, as shown in **Figure 2**. MDPNet consists of two parallel paths designed to efficiently genotype gliomas using multimodal MRI data. The model is composed of two main components: attention-guided asymmetric dual-path feature extraction (ADFE) and gene typing guided by inter-gene relationships(IGT).

ADFE primarily employs two parallel pathways to extract and enhance feature information from input multi-modal MRI data. By utilizing strategies of feature fusion and residual connections, this approach particularly strengthens the heterogeneous features associated with glioma lesions in T2 and FLAIR modalities. Consequently, it enhances the network’s capability to discern subtle differences in the structural characteristics of glioma lesion tissues. Subsequently, by employing various attention mechanisms to extract distinct features from the enhanced data, global contextual information can be captured to address the limitations of CNN, while also extracting more discriminative high-order feature information, thereby providing essential conditions for accurate classification in future analyses. IGT leverages the higher-order feature information provided by ADFE and the inter-gene relationships to construct a pathway for guiding the 1p/19q classification using the genotyping results of the IDH and ATRX genes. This strategy allows for the simultaneous prediction of IDH mutation status, 1p/19q codeletion status, and ATRX mutation status, while also significantly enhancing the network’s typing ability for the three gene molecules. Detailed descriptions of these two components are provided in the following sections.

### 2.1 Attention-guided asymmetric dual-path feature extraction (ADFE)

Currently, research on gene molecular typing of gliomas typically employs a singular approach to extract feature information from multimodal MRI data. Although this method accounts for the heterogeneity of gliomas and is advantageous for gene molecular typing, it overlooks the impact of mismatched features between T2 and FLAIR modalities on gene molecular typing, particularly regarding IDH. This study proposes an attention-guided asymmetric dual-path feature extraction method (ADFE) designed to extract multimodal MRI features while thoroughly exploring and enhancing the heterogeneity of T2 and FLAIR modalities, thus further improving the performance of gene molecular typing.

Specifically, ADFE comprises two parallel feature extraction paths. The first path comprehensively processes the fused feature information from four modalities: T1, T1-ce, T2, and FLAIR. The second path focuses on extracting mismatched features between the T2 and FLAIR modalities. Feature enhancement is performed in the middle to enable information interaction between the two paths, thereby enhancing the heterogeneous feature information from the T2 and FLAIR modalities, ultimately improving the prediction performance of the network.

#### 2.1.1 Enhancement of T2 and FLAIR feature information

In ADFE, the first path initially performs a 3 × 3 × 3 convolution operation on the fused images of four MRI modalities, generating a feature map with 16 channels. To capture spatial and feature representations, a feature map is generated through multiple stacked residual convolution blocks and downsampling operations, producing a feature map $F\in {\mathbb{R}}^{C\times H\times W\times D}$ . Each residual convolution block consists of two convolutional layers with kernel sizes of 3×3×3. For the downsampling operation, a 3×3×3 convolution with a stride of 2 is used instead of pooling, and the output channel is set to twice the input channel. The second path, based on 3D ResNet18, begins by performing an initial convolution on the fused images of two MRI scans. The resulting feature maps are then processed through multiple stacked residual convolution blocks with kernel sizes of 3 × 3 × 3 to capture feature information.

After extracting feature information from each input image through both paths, the network fuses the low-level feature information from the two paths. This step achieves information exchange between the two paths through concatenation and convolution of the initially extracted low-level features. After passing through the residual convolution blocks of both paths and the downsampling operation with a kernel size of 3 × 3 × 3 and a stride of 2 in the first path, feature maps *F*_1_ and *F*_2_ with 128 channels and dimensions of 16 × 16 × 16 are obtained, respectively. The feature maps *F*_3_ and *F*_4_ are then concatenated along the channel dimension to produce a feature map *F*_5_ of size 16 × 16 × 16 with 256 channels. Subsequently, this feature map is integrated and its dimensionality is reduced through a convolution with a kernel size of 1×1×1, reducing the number of channels to 128. Finally, the reduced feature information is fused with the previously extracted feature information via a residual connection to enhance feature representation. This feature enhancement process not only retains all the feature information from both paths but also promotes the complementarity and enhancement of mismatched features between the T2 and FLAIR modalities. The 1 × 1 × 1 convolution operation promotes deep feature fusion while controlling model complexity through channel dimensionality reduction, thus reducing the risk of overfitting and maintaining the network’s ability to distinguish glioma molecular typing tasks. Through this process, effective information exchange is achieved between the two paths, enhancing key lesion features relevant to both.

**Figure 3:**
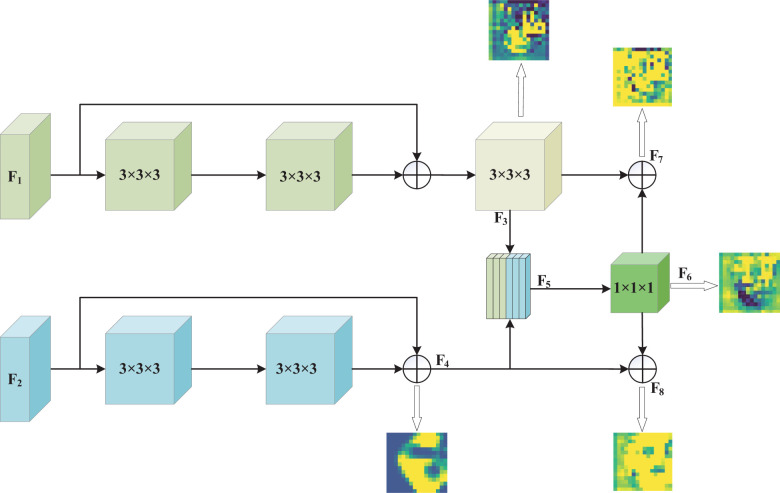
The feature enhancement process of T2 and FLAIR modalities by MDPNet.

**Figure 4:**
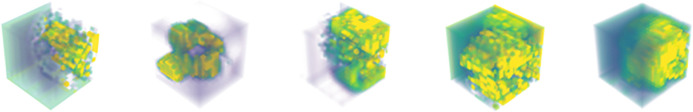
The 3D feature maps of MDPNet at different stages during the feature enhancement process for T2 and FLAIR modalities, from left to right, represent one channel of the 3D feature maps *F*_3_, *F*_4_, *F*_6_, *F*_7_, and *F*_8_, respectively.

To demonstrate that our method effectively enhances key features of glioma lesion areas, we used the lesion area from the first row of the MR image in **Figure 1** as the center and cropped the image data to a size of 128 × 128 × 128 for input into the MDPNet model. In **Figure 3**, we presented 2D slices of the 16 × 16 × 16 feature maps generated by MDPNet at different training stages. The yellow regions in the feature maps represent the highly focused areas of the network, and the expansion of these yellow regions corresponds to the enhanced ability of the network to capture key features of lesion areas. To further demonstrate the effectiveness of our proposed method, we visualized a channel of the 3D feature map obtained at different stages during the feature enhancement process in **Figure 3**. The visualized 3D feature map is shown in **Figure 4**.

From **Figures 3**, **4**, it can be observed that our method improves the network’s attention to the glioma lesion area, providing more accurate and detailed feature inputs for subsequent high-order feature extraction and gene typing.

#### 2.1.2 Deep feature information extraction

Convolutional neural networks(CNN) excel at extracting local features and structural information from data, while the attention mechanism dynamically assigns different weights to the features extracted by the CNN, emphasizing important feature information and downplaying irrelevant parts. Therefore, combining the two can enhance the learning and utilization efficiency of the model for key features (21, 22).

To improve the representation of high-order features, the latter half of ADFE employs the attention mechanism. In the first path, we used the Vision Transformer (ViT) (23). For three-dimensional volume data, we extended the ViT model to handle 3D data by partitioning the data into 3D blocks. However, embedding large-sized 3D patches inevitably increases the computational overhead of transformer. To address this issue, the low-resolution feature map $F_{\text{λ}}\in {\mathbb{R}}^{128\times 16\times 16\times 16}$ , extracted from the CNN, is fed into the Vision Transformer to learn global feature representations. To ensure comprehensive and in-depth feature representation for each 3D volume, we utilized a linear projection layer composed of convolutions with kernel sizes of 3 × 3 × 3. This layer expands the number of channels in the feature map from 128 to 512, thereby enhancing its expressive power. Subsequently, to provide a more compact and efficient input for the model, we reshaped the adjusted feature maps into input tokens for the Vision Transformer. To encode positional information, learnable position embeddings *E_pos_* are integrated into patch embeddings *E_pat_* through addition operations, resulting in the final feature embedding *Z*. Then, *Z* is fed into 4 stacked Transformer layers. Each transformer layer consists of Multi-Head Self-Attention(MSA) and a multi-layer perceptron block. The self-attention input at layer l is as shown in Equation 1:


(1) $$Q=Z_{l-1}W_{Q},K=Z_{l-1}W_{K},V=Z_{l-1}W_{V}$$

where *W_Q_*,*W_K_*,*W_V_* are learnable parameters of three linear projection layers. The self-attention computation is as shown in Equation 2:


(2) $$SA\;\left(Z^{l-1}\right)=Softmax\;\left(\frac{QK^{T}}{\sqrt{d}}\right)V$$

where *d* is the dimension of the triplet (*Q,K,V)*. The output of the multi-head self-attention module is then transformed by a multi-layer perceptron block with residual connections, serving as the output of that layer.

**Figure 5:**
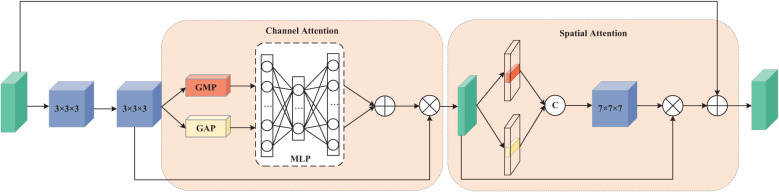
3D Residual Dual Attention Module (3D-RDA).

The first pathway is designed to process data from four MRI modalities: T1, T1ce, T2, and FLAIR. This requires the network to capture global dependencies to establish an accurate contextual representation. Therefore, we adopt a Vision Transformer (ViT) architecture, as ViT excel at capturing global information. In contrast, the second pathway focuses on learning the mismatched features between T2 and FLAIR modalities. These mismatches typically manifest as local rather than global variations, necessitating a network with strong local feature extraction capabilities. To achieve this while maintaining computational efficiency, we constructed a 3D Residual Dual Attention Module (3D-RDA), as shown in **Figure 5**. This module extracts high-order features from the enhanced features by sequentially embedding channel attention and spatial attention in each residual block of 3D ResNet18. This design aims to refine features through the residual architecture while utilizing attention mechanisms to enhance the model’s ability to identify and extract key information. 3D-RDA takes feature map *F*_8_ as input.

In the channel attention section, the input $M\in {\mathbb{R}}^{C^{\prime }\times H^{\prime }\times W^{\prime }\times D^{\prime }}$ is processed through a series of transformations to obtain a three-dimensional channel attention map, generating channel features $M^{\prime }\in {\mathbb{R}}^{C^{\prime }\times H^{\prime }\times W^{\prime }\times D^{\prime }}$ . The channel attention mechanism promotes effective feature differentiation and enhancement by learning the interdependence between channels, thereby improving the feature representational power (24). The output of this part, $U\in {\mathbb{R}}^{C^{\prime }\times H^{\prime }\times W^{\prime }\times D^{\prime }}$ , is as shown in Equation 3:


(3) UM=σ(MLP(AvgPool(M))+MLP(MaxPool(M)))×M

where *σ* is the sigmoid activation function. The spatial attention part focuses on the spatial information of the glioma lesions. The input of this part is *U*, and the output $U^{\prime }\in {\mathbb{R}}^{C^{\prime }\times H^{\prime }\times W^{\prime }\times D^{\prime }}$ , is as shown in Equation 4:


(4) $$U^{\prime }\left(U\right)=\sigma \left(Conv\left(\left[AugPool\left(U\right);MaxPool\left(U\right)\right]\right)\right)\times U$$

where *Conv* represents a 7 × 7 × 7 3D convolution. Residual connections and feature fusion then perform element-wise addition of the input feature map $F^{\prime }$ and the spatial attention output $U^{\prime }$ , yielding output $F^{\prime }+U^{\prime }$ . Embedding these two attention mechanisms into each residual structure greatly enhances the model’s ability to capture glioma lesion features.

By extracting differentiated features from the enhanced features, our model can thoroughly capture and learn the lesion characteristics of gliomas from multiple perspectives. This strategy, which integrates various information processing methods, has further strengthened the model’s foundational performance and improved the network’s ability to classify gene molecules. In summary, ADFE significantly enhances the network’s ability to capture key lesion features of gliomas through a carefully designed dual-path parallel fusion architecture and feature fusion strategy, providing more precise and enriched feature inputs for subsequent gene typing. It is a core component for achieving efficient molecular typing.

### 2.2 Gene typing guided by inter-gene relationships (IGT)

In the study of molecular subtyping of glioma genes, the task of multi-gene joint subtyping often treats each gene as an independent unit for individual predictions. This approach overlooks the complex interactions between genes and fails to fully utilize their relationships, thereby limiting the model’s predictive performance. To address this limitation, we designed a gene typing guided by inter-gene relationships(IGT) strategy in this study. This strategy leverages weight sharing between different genes and utilizes inter-gene relationships to improve the overall performance of the network, particularly enhancing its performance in the classification of 1p/19q.

Regarding the interrelationships among the IDH, ATRX, and 1p/19q genetic markers, in brief: when the IDH gene is wild-type, the 1p/19q is typically in a non-codeletion state; when the ATRX gene is mutant, the 1p/19q gene is also generally non-codeletion. However, these relationships are unidirectional and not absolute, meaning that the state information of a single genetic marker is insufficient to directly infer the specific status of other markers (25, 26). Therefore, we propose simultaneously classifying these three genetic markers. In our model architecture, we designed two auxiliary paths: one guides the classification of 1p/19q based on the IDH classification results, and the other influences 1p/19q classification through the ATRX classification results.

Specifically, after extracting advanced features from the input image using ADFE, we fuse the features at different scales to construct a multi-scale classification network for the three gene molecular subtypes. We use global average pooling(GAP) and global max pooling(GMP) to transform the multi-scale feature maps from each channel into a uniform size. GAP and GMP were chosen because they do not require any additional parameters for optimization. These collected multi-scale features are then fused via concatenation and input into a fully connected layer consisting of 1792, 1280, 512, 32, and 2 neurons. Finally, the softmax function outputs the probabilities of the three gene molecular subtypes. The probability representation is as shown in Equations 5–7:


(5) $$P_{idh}^{\left(i\right)}=Softmax\left(N_{idh}\right)$$

(6) $$P_{atrx}^{\left(i\right)}=Softmax\left(N_{atrx}\right)$$

(7) $$P_{1p/19q}^{\left(i\right)}=Softmax\left(Concat\left[N_{1p/19q};\max\left(P_{idh}^{\left(i\right)}\right);\max\left(P_{atrx}^{\left(i\right)}\right)\right]\right)$$

where *N* represents the fully connected network for gene typing, *Concat* represents the concatenation operation, and $P^{\left(i\right)}\in {\mathbb{R}}^{1\times 2}$ is the output probability of sample *i* belonging to a certain category.

The design of IGT aims to leverage the known typing information of IDH and ATRX to guide the determination of 1p/19q gene status, thereby enhancing the model’s ability to utilize interdependencies between genes, improving the overall performance of the network, and addressing the shortcomings of traditional gene typing methods. Moreover, this strategy mitigates the issue of biased predictions that arise during training due to the severe class imbalance between the 1p/19q non-codeletion and codeletion subtypes, ultimately enhancing the model’s accuracy in distinguishing 1p/19q genetic subtypes.

The subsequent ablation experiments in this study will validate the effectiveness of our proposed method, revealing the specific contributions and limitations of each gene to the final typing performance through comparative analysis.

### 2.3 Loss function

The issue of class imbalance is a significant challenge that cannot be overlooked in the analysis of medical imaging data. In our study, the number of IDH wild-type cases is approximately twice that of IDH mutant cases, highlighting the uneven data distribution. Furthermore, among the 241 samples, ATRX mutant and 1p/19q codeleted cases account for only 20% (48 cases) and 11% (27 cases), respectively. The severe imbalance in sample categories not only poses a significant challenge to the model’s classification accuracy but also may lead to overfitting toward the majority class, thus neglecting the learning of minority class features.

To address this challenge, we adopted a strategy by introducing large margin aware focal(LMF) loss (27) to mitigate the class imbalance problem in medical imaging. LMF loss jointly optimizes focal loss and LDAM loss, aiming to alleviate the negative impact of class imbalance by dynamically adjusting the loss weights. This ensures that the model can significantly improve its detection accuracy for minority classes while efficiently classifying the majority class. This loss function is as shown in Equation 8:


(8) $$ℒ=\alpha {ℒ}_{LDAM}+\beta {ℒ}_{FL}$$

where ${ℒ}_{LDAM}$ and ${ℒ}_{FL}$ are the LDAM Loss (28) and Focal Loss (29), respectively, and *α* and *β* are two hyperparameters. The calculations for Focal Loss and LDAM Loss are shown in Equations 9, 10:


(9) $${ℒ}_{FL}\left(p_{t}\right)=-\alpha_{t}{\left(1-p_{t}\right)}^{\gamma }\text{ log}\left(p_{t}\right)$$

where $p_{t}$ denotes the probability of correct classification by the model, $\alpha_{t}$ is the weight of class *t*, and *γ* is a modulation factor.


(10) $${ℒ}_{LDAM\;}=-\sum_{i-1}^{c}y_{i}\cdot \;\frac{1}{1-e^{-m\left(max\left(0,\;m_{i}-\delta \right)+1\right)}}\cdot \text{log}\left(p_{i}\right)$$

where $y_{i}$ represents the label of class *i*, *p_i_* is the probability predicted by the model for class *i*, *m* is a predefined hyperparameter, *m_i_* represents the margin for class *i* (with smaller values for more frequent classes), and *δ* is a threshold. In our study, as the typing of the three gene molecules is combined, improper task weight settings can lead to network bias, causing the network to focus more on predicting one specific gene molecule. To mitigate this negative impact, we designed a method to adaptively adjust the weights for the loss functions of the three gene molecular typings. This loss function is as shown in Equation 11:


(11) $$\begin{array}{c} {ℒ}_{mul}=\frac{1}{3\sigma_{idh}^{2}}{ℒ}_{idh}+\frac{1}{3\sigma_{1p/19q}^{2}}{ℒ}_{1p/19q}+\frac{1}{3\sigma_{atrx}^{2}}{ℒ}_{atrx}\\ +\log(\sigma_{idh}\sigma_{1p/19q}\sigma_{atrx}) \end{array}$$

where ${ℒ}_{idh}$ , ${ℒ}_{1p/19q}$ , and ${ℒ}_{atrx}$ are the LMF Loss used for the typing of IDH, 1p/19q, and ATRX gene molecules, respectively. $\sigma_{idh}$ , $\sigma_{1p/19q}$ , and $\sigma_{atrx}$ are uncertainty weights and learnable parameters during network training. Initially, $\sigma_{idh}$ , $\sigma_{1p/19q}$ , and $\sigma_{atrx}$ are initialized to tensors with values 5.0, 6.0 and 6.0, respectively, and are iteratively updated adaptively during the training phase.

## 3 Experiments

### 3.1 Datasets

In the field of medical image processing, the scarcity of data has severely constrained the progress of related research. Due to limited data availability, researchers face challenges in conducting comprehensive studies. In our study, we utilized two datasets: (1)BraTS2020, which is a publicly available dataset that initially included 494 independent glioma cases, officially recommended to be divided into 369 training cases and 125 validation cases. By integrating gene status information obtained from the TCGA database and performing data cleaning, we ultimately compiled a dataset of 241 cases with both imaging and molecular data. Following the official recommended split, these cases were divided into a training set (166 cases) and a validation set (75 cases) for model training and performance evaluation. Detailed data statistics are shown in **Table 1**. (2) A private dataset from the General Hospital of Ningxia Medical University, which contains data from 95 patients from the Department of Neurosurgery of the General Hospital of Ningxia Medical University between January 2023 and June 2024,with detailed data statistics shown in **Table 2**.

**Table 1:** Dataset statistics for genotyping experiments.

Statistics	Training data	Validation data
Subject n	166	75
Age median (range)	54 (18-84)	55 (23-80)
Sex		
Male	Male	Male
Female	Female	Female
Unknown	Unknown	Unknown
IDH status		
Mutant	Mutant	Mutant
WT	WT	WT
ATRX status		
Mutant	Mutant	Mutant
WT	WT	WT
1p/19q status		
Codel	Codel	Codel
Non-codel	Non-codel	Non-codel

**Table 2:** Internal dataset statistics.

Genetic subtype	IDH	ATRX	1p/19q
Mutant (codel)	49 [51.6%]	67 [70.5%]	15 [15.8%]
WT (no-codel)	46 [48.4%]	28 [29.5%]	80 [84.2%]

For the public dataset, we utilized all four imaging modalities, namely T1, T1-ce, T2, and FLAIR. The fused images of these four modalities were used as input for our model. In clinical practice, due to the limited number of patients who undergo full modality imaging, most patients have incomplete modality imaging. In our private dataset, the majority of patients possess only a subset of modalities, typically T1, T2, and FLAIR, or T1-ce, T2, and FLAIR. To address this issue, we employ a “Zero Padding” strategy during the initial fusion stage, where missing modalities are replaced with zero-filled input matrices. This approach ensures that all input data maintain a consistent dimensionality across samples, enabling the network to process various modality combinations while mitigating data loss that would otherwise occur if incomplete samples were discarded. A detailed statistical summary of the available MRI modalities within our internal dataset is provided in **Table 3**.

**Table 3:** Detailed statistics of available MRI modalities for all patients in the internal dataset.

Modalities combination	Quantity	Percentage
T1+T1ce+T2+FLAIR	16	16.8%
T1+T2+FLAIR	47	49.5%
T1ce+T2+FLAIR	32	33.7%

For our own dataset, each patient underwent pathological and immunohistochemical (IHC) examinations to confirm the glioma type and its corresponding genotype, and was assessed based on preoperative MRI scans. All selected patients met the following inclusion criteria: (1) age greater than 18 years; (2) histopathologically confirmed diagnosis of glioma; (3) preoperative MRI scans; (4) availability of IDH, ATRX, and 1p/19q genetic status results. Specifically, pathologists used immunohistochemistry (IHC) to detect mutations in the IDH and ATRX genes, and fluorescence *in situ* hybridization (FISH) to determine if the short arm of chromosome 1 and the long arm of chromosome 19 were deleted. This study has received ethical approval.

For these 95 patients’ data, we first used the dicom2nifti package in Python to convert DICOM-format MR images into NIFTI format. Then, we used the SPM package in Matlab (https://www.fil.ion.ucl.ac.uk/spm/ ) to register the NIFTI files to a uniform resolution of 1mm³. Finally, we applied FSL software (https://fsl.fmrib.ox.ac.uk/fsl ) to perform skull stripping on the registered MR images.

### 3.2 Implementation details

#### 3.2.1 Data preprocessing

Before training and testing the model, the first critical step involves the fusion of multi-modal imaging data. This fusion process enables the network to effectively capture inter-modal associations, thereby enhancing its ability to comprehend and model multi-modal information. During the fusion process, the Z-score normalization method is first applied to adjust the pixel values, and the images from each modality are processed to a size of 240×240×155 voxels. The standardized 3D volumes from all modalities are then stacked along the last dimension to form a unified multi-modal representation. This fusion strategy not only preserves the unique information from each modality but also facilitates the effective integration of multi-modal information.

Given the insufficient number of samples and the extreme imbalance in the sample distribution across different classes, this could potentially lead to overfitting or underfitting during training, which would diminish the model’s generalization ability. Therefore, in order to provide more training samples, the fused images undergo random cropping and flipping operations. Specifically, the original 240×240×155 images are randomly cropped to 128×128×128 voxels, which are subsequently used as inputs to the network.

#### 3.2.2 Experimental setup and evaluation metrics

In this study, all model training and ablation studies were conducted on a NVIDIA GeForce RTX 3090 GPU with 24GB of memory. The model was optimized using the Adam optimizer, with an initial learning rate set to 1e-4 and a weight decay of 1e-5. The batch size was configured to 2, and the total number of epochs was set to 1000. The final performance metrics were derived from the epoch in which the validation loss reached its minimum across the 1000 training iterations.

To evaluate the performance of the model, we used several metrics: area under the curve (AUC), accuracy (Acc), sensitivity (Sens), and specificity (Spec) for the quantitative evaluation of genetic subtyping.

## 4 Results

### 4.1 Ablation experiments

#### 4.1.1 Importance analysis of T2 and FLAIR modalities

To verify the impact of mismatched features between the T2 and FLAIR modalities on genetic subtyping, as well as the necessity of the second path, we conducted an ablation study on the second path and its input modalities. While the first pathway utilized all four modalities as input, the second pathway was separately fed with T2, FLAIR, and a fusion of these two modalities. The experimental results are shown in **Table 4**. As observed, the network’s ability to classify the three genetic markers was the weakest when only the first pathway was used. In contrast, the best classification results were achieved when the second pathway utilized the fused T2 and FLAIR image data. Among the modalities, T2 had the most significant impact on the classification of 1p/19q, while FLAIR had the greatest influence on the classification of IDH. The experimental results indicate that the T2 and FLAIR modalities contain significant heterogeneous information related to the IDH and 1p/19q molecular status. By enhancing the network’s learning of key glioma features in the T2 and FLAIR modalities, the model’s precision in molecular classification tasks was effectively improved. This further confirms the effectiveness and rationality of the proposed strategy.

**Table 4:** Ablation experiment on the importance of T2 and FLAIR modalities.

T2	FLAIR	DH				ATRX				1p/19q			
		Acc	AUC	Sens	Spec	ACC	AUC	Sens	Spec	ACC	AUC	Sens	Spec
		84.0%	0.86	**84.4%**	83.7%	82.7%	0.82	53.3%	90.0%	81.3%	0.75	0	**100%**
✓		81.3%	0.86	59.4%	**97.7%**	85.3%	0.80	33.3%	**98.3%**	85.3%	0.75	21.4%	**100%**
	✓	**86.7%**	**0.91**	81.3%	90.7%	85.3%	0.82	66.7%	90.0%	82.7%	0.79	14.3%	98.4%
✓	✓	**86.7%**	0.90	75.0%	95.3%	**92.0%**	**0.88**	**80.0%**	95.0%	**89.3%**	**0.80**	**42.9%**	**100%**

✓: Indicates whether this modality is used in the second path.

Bold values: Indicates the best result for the current metric.

#### 4.1.2 Analysis of the effectiveness of heterogeneous attention mechanisms

To investigate the impact of employing different attention mechanisms in the upper and lower pathways on model performance, we designed an ablation study that evaluates various attention mechanism configurations. The experimental results are summarized in **Table 5**, which includes four configurations: (1) both pathways utilizing 3D-RDA(All_3D-RDA); (2) both pathways adopting the Vision Transformer(All_ViT); (3) a hybrid model where the first pathway employs 3D-RDA and the second pathway utilizes ViT(3D-RDA+ViT); and (4) the proposed architecture in this study, where the first pathway adopts ViT and the second pathway employs 3D-RDA. As shown in the table, our proposed network architecture achieves the best performance in genetic subtyping. This finding suggests that employing ViT in the first pathway effectively captures long-range dependencies in the input data, thereby enhancing the model’s ability to recognize and utilize global contextual information. Meanwhile, integrating 3D-RDA into the second pathway strengthens the model’s capacity for local feature representation, allowing it to extract heterogeneous features between the T2 and FLAIR modalities more effectively. This synergistic combination enhances the accuracy of glioma genetic subtyping.

**Table 5:** Ablation study on heterogeneous attention mechanisms.

Combination of different attention mechanisms	IDH				ATRX				1p/19q			
	ACC	AUC	Sens	Spec	ACC	AUC	Sens	Spec	ACC	AUC	Sens	Spec
All_3D-RDA	84.0%	0.86	**84.4%**	83.7%	82.7%	0.85	53.3%	90.0%	81.3%	0.75	0	**100%**
All_ViT	81.3%	0.86	59.4%	**97.7%**	85.3%	0.80	33.3%	**98.3%**	85.3%	0.75	21.4%	**100%**
3D-RDA+ViT	**86.7%**	**0.91**	81.3%	90.7%	85.3%	0.82	66.7%	90.0%	82.7%	0.79	14.3%	98.4%
MDPNet	**86.7%**	0.90	75.0%	95.3%	**92.0%**	**0.88**	**80.0%**	95.0%	**89.3%**	**0.80**	**42.9%**	**100%**

Bold values: Indicates the best result for the current metric.

#### 4.1.3 Analysis of the impact of IDH and ATRX on 1p/19q gene molecular typing

To validate the effectiveness of the IGT strategy, we performed ablation experiments, with the results shown in **Table 6**. The experiments involved progressively removing the IDH or ATRX auxiliary paths to quantify their direct impact on overall classification performance. The data analysis indicates that incorporating the classification information of IDH and ATRX significantly enhances the 1p/19q classification accuracy as well as the classification performance of IDH and ATRX themselves. This directly confirms the effectiveness of the IGT strategy. This strategy not only optimizes the model’s classification performance but also effectively addresses the prediction bias caused by data imbalance, reinforcing the model’s practicality and accuracy in glioma gene molecular classification tasks. This strategy provides a new perspective for deep learning-assisted precision medicine research.

**Table 6:** Ablation study on the impact of IDH and ATRX on 1p/19q genetic molecular subtyping.

IDH	ATRX	IDH				ATRX				1p/19q			
		ACC	AUC	Sens	Spec	ACC	AUC	Sens	Spec	ACC	AUC	Sens	Spec
		81.30%	**0.90**	68.8%	90.7%	88.0%	0.84	46.7%	**98.3%**	86.7%	0.79	28.6%	**100%**
	✓	82.70%	0.85	75.0%	88.4%	88.0%	**0.92**	60.0%	95.0%	85.3%	0.70	28.6%	**100%**
✓		85.30%	**0.90**	**81.3%**	88.4%	88.0%	0.87	73.3%	91.7%	85.3%	**0.80**	21.4%	**100%**
✓	✓	**86.70%**	**0.90**	75.0%	**95.3%**	**92.0%**	0.88	**80.0%**	95.0%	**89.3%**	**0.80**	**42.9%**	**100%**

✓: Indicates whether incorporating the genotyping results of this gene during classification has an impact on the 1p/19q molecular genotyping.

Bold values: Indicates the best result for the current metric.

### 4.2 Comparison with the state-of-the-art methods

**Figure 6:**
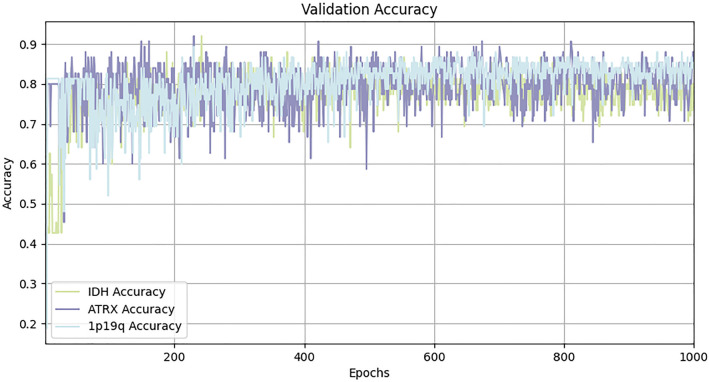
Accuracy curve of MDPNet on validation set.

To demonstrate the superiority of our proposed method, we compared it with various existing classification methods using the BraTS2020 dataset, with the experimental results presented in **Table 7**. MDPNet exhibited promising performance across key evaluation metrics, including accuracy, AUC, sensitivity, and specificity. Specifically, the classification accuracies for IDH, ATRX, and 1p/19q reached 86.7%, 92.0%, and 89.3%, respectively. Compared to existing methods, MDPNet achieved an average improvement of 5.4% in IDH prediction accuracy, 6.2% in ATRX prediction accuracy, and 13.7% in 1p/19q prediction accuracy. The accuracy curve of our model on the validation set is shown in **Figure 6**. To better showcase the performance of our network, we also conducted experiments on several commonly used baseline networks in addition to the comparison with state-of-the-art models. The experimental results indicated that our method significantly outperformed these baseline networks. Simultaneous typing of the three gene markers can not only assist doctors in determining the type of glioma preoperatively but also help reduce the high costs associated with genetic testing for patients.

**Table 7:** Comparative experiments with the state-of-the-art models.

Models	IDH				ATRX				1p/19q			
	ACC	AUC	Sens	Spec	ACC	AUC	Sens	Spec	ACC	AUC	Sens	Spec
MTTU-Net (17)	84.0%	0.86	**84.4%**	87.3%	82.7%	0.82	53.3%	90.0%	81.3%	0.65	0	**100%**
DST (30)	78.7%	0.85	50.0%	**100%**	88.0%	0.86	73.3%	91.7%	73.3%	0.66	35.7%	**82.0%**
PRIYANKA (31)	84.0%	0.89	78.1%	88.4%	84.0%	0.80	40.0%	91.7%	81.3%	0.71	0	100%
Sebastian (32)	84.0%	0.85	75.0%	90.7%	84.0%	**0.88**	73.3%	86.7%	82.7%	0.76	14.3%	98.4%
Shi (33)	81.3%	**0.90**	59.4%	97.7%	88.0%	0.86	73.3%	91.7%	85.3%	**0.80**	35.7%	96.7%
M3D-DenseNet (34)	76.0%	0.83	46.9%	97.7%	88.0%	0.85	73.3%	91.7%	70.7%	0.61	28.6%	80.3%
Resnet50 (35)	78.7%	0.85	50.0%	**100%**	88.0%	0.86	73.3%	91.7%	73.3%	0.66	35.7%	82.0%
Densenet121 (36)	77.3%	0.86	46.9%	**100%**	84.0%	0.81	60.0%	90.0%	74.7%	0.66	35.7%	83.6%
Senet101 (24)	82.7%	0.87	59.4%	**100%**	78.7%	0.77	60.0%	83.3%	74.7%	0.71	**50.0%**	80.3%
**MDPNet**	**86.7%**	**0.90**	75.0%	95.3%	**92.0%**	**0.88**	**80.0%**	**95.0%**	**89.3%**	**0.80**	42.9%	**100%**

Bold values: Indicates the best result for the current metric.

### 4.3 Performance of MDPNet on internal dataset

The internal dataset used in this study was treated as an independent test set to evaluate the generalization capability of the proposed MDPNet. The accuracy of the test results are presented in **Table 8**. The experimental results indicate that, although only three modalities from the internal dataset were used, MDPNet still demonstrated excellent and stable performance, particularly in its ability to predict ATRX, achieving an accuracy of 92.6%. These findings indicate that MDPNet has robust stability and generalizability to predict the statuses of IDH, ATRX, and 1p/19q.

**Table 8:** Test results of MDPNet on internal datasets.

Datasets	IDH				ATRX				1p/19q			
	ACC	AUC	Sens	Spec	ACC	AUC	Sens	Spec	ACC	AUC	Sens	Spec
BraTS2020	86.7%	0.90	75.0%	95.3%	92.0%	0.88	80.0%	95.0%	89.3%	0.80	42.9%	100%
Internal	80.0%	0.81	75.5%	84.8%	92.6%	0.85	92.5%	92.9%	84.2%	0.80	33.3%	93.8%

### 4.4 Prognostic analysis of IDH and ATRX genotypes

**Figure 7:**
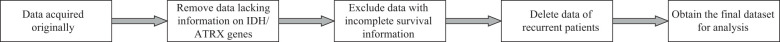
Workflow for the selection of prognostic analysis data for IDH and ATRX.

To investigate the clinical relevance of molecular subtypes, we performed Kaplan-Meier survival analysis using clinical data from 887 glioma patients sourced from the TCGA-LGG and TCGA-GBM datasets. These data include genetic subtyping information, patient age, sex, survival time, and follow-up records. After applying the selection criteria outlined in **Figure 7**, a total of 598 cases were included in the prognostic analysis for IDH, while 390 cases were available for ATRX prognostic evaluation. Detailed statistics are provided in **Table 9**. Since the survival data were recorded in days, we adjusted them to a standardized monthly scale by grouping every 30 days during the Kaplan-Meier survival analysis.

**Table 9:** Dataset statistics for prognostic analysis of IDH and ATRX.

Statistics	LGG (IDH)	GBM (IDH)	LGG (ATRX)	GBM (ATRX)
WT	52	471	76	256
Mutant	66	9	40	18
Total	118	480	116	274

**Figure 8:**
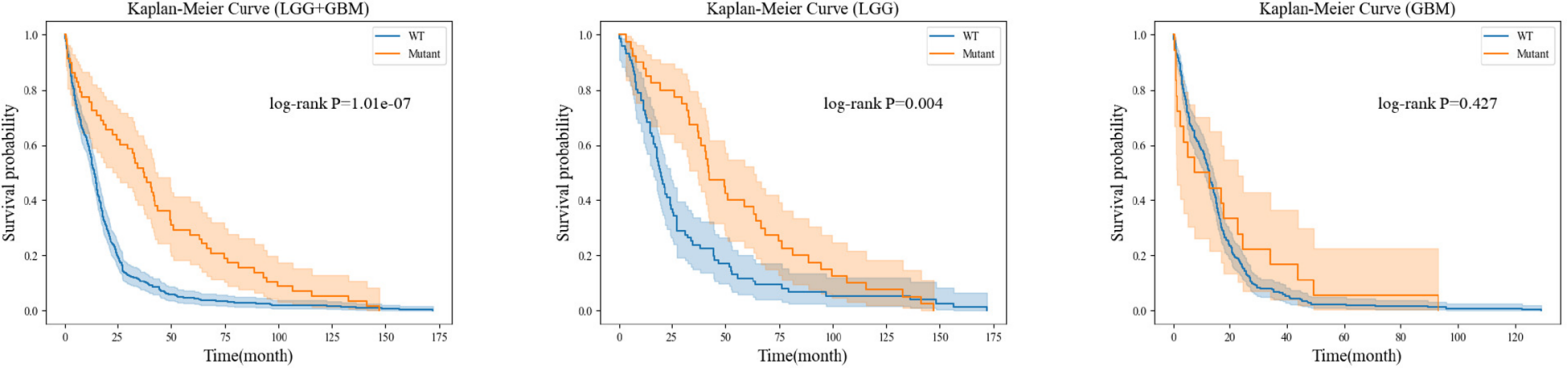
Prognostic analysis based on different genetic states of ATRX.

**Figure 9:**
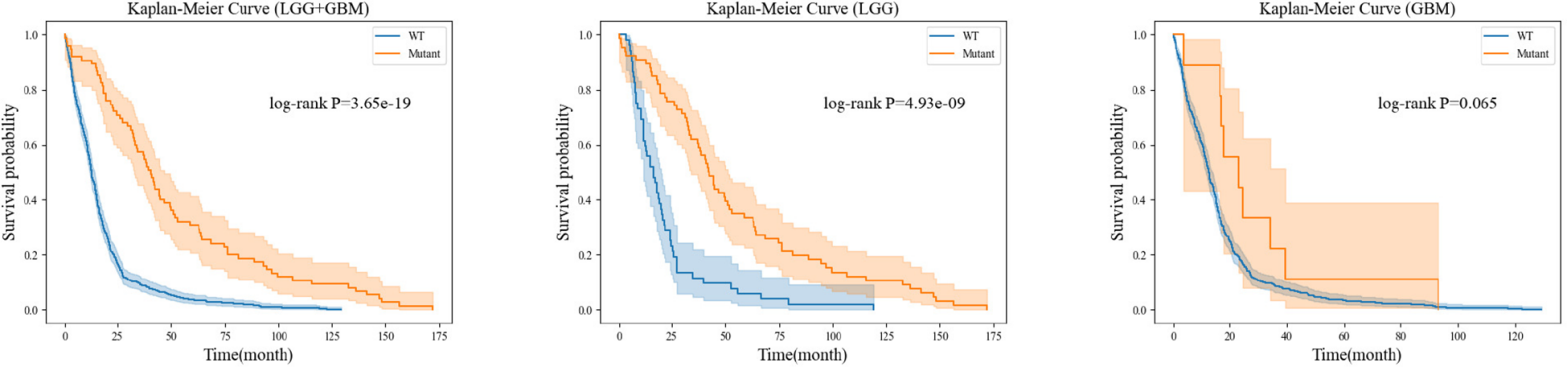
Prognostic analysis based on different genetic states of IDH.

For each gene molecule, we conducted three experiments based on the grade of glioma, including the low-grade glioma group (LGG), high-grade glioma group (GBM), and a mixed group of LGG and GBM (LGG+GBM). The prognostic experiment for the ATRX gene molecule is shown in **Figure 8**.The prognostic experiment for the IDH gene molecule is shown in **Figure 9**.

Kaplan-Meier analysis revealed that patients with ATRX mutations exhibited significantly longer survival than those with wild-type ATRX in the LGG group (*p* = 0.004) and the mixed group (*p <* 0.001). Similarly, IDH-mutant patients demonstrated a markedly improved survival prognosis compared to their wild-type counterparts in both the LGG group (*p <* 0.001) and the mixed group (*p <* 0.001). These findings are consistent with existing clinical evidence, further reinforcing the importance of precise preoperative genetic subtyping. Early molecular characterization not only facilitates timely therapeutic decision-making for clinicians but also reduces the financial burden of expensive genetic testing for certain patients. Ultimately, accurate genetic stratification plays a crucial role in guiding prognosis assessment and optimizing subsequent treatment strategies.

## 5 Discussion

In this study, we propose a Dual-Path Parallel Fusion Network (MDPNet) for multimodal MRI-based glioma genotyping, specifically targeting three critical genetic biomarkers: IDH, 1p/19q, and ATRX. Experimental results demonstrate that MDPNet achieves superior performance on public datasets, effectively predicting these molecular subtypes in glioma patients. This methodology shows potential for assisting clinicians in preoperative assessment of glioma molecular profiles. Compared with existing approaches, MDPNet demonstrates significant improvements across multiple performance metrics.

The enhanced performance stems from two principal innovations: (1) A dual-branch parallel architecture enabling efficient integration of multimodal MRI features, particularly emphasizing the extraction of heterogeneous characteristics between T2 and FLAIR sequences. This design prioritizes discriminative feature learning from these critical modalities. (2) A novel classification framework incorporating crossgeneic feature sharing and molecular interrelationships, complemented by specialized loss functions. This approach not only improves 1p/19q classification accuracy and mitigates prediction bias during model training, but also enhances IDH and ATRX subtyping capabilities. Ablation studies confirm that leveraging T2-FLAIR heterogeneity significantly boosts genotyping performance for all three biomarkers. Furthermore, the utilization of inter-molecular relationships proves instrumental in optimizing model efficacy.

In this study, we integrated multi-modal MRI data into the dual-branch architecture of our model, MDPNet, and compared its performance with state-of-the-art methods such as MTTU-Net and DST. Unlike these approaches, MDPNet places greater emphasis on extracting the heterogeneity between T2 and FLAIR modalities while incorporating a gene relationship-guided classifier and an optimized loss function. As a result, our model demonstrated significant performance improvements, achieving the highest accuracy and AUC scores across all key metrics. Specifically, the classification accuracy and AUC values for IDH, ATRX, and 1p/19q reached 86.7% and 0.90, 92.0% and 0.88, and 89.3% and 0.80, respectively. Compared to widely used deep learning models such as ResNet50, DenseNet121, and SENet101, MDPNet outperformed them by a substantial margin, with accuracy improvements of 7.1% for IDH, 8.4% for ATRX, and 15.1% for 1p/19q. These results indicate that MDPNet achieves superior classification precision and more reliable predictions. In addition to training and validation on the public BraTS2020 dataset, we further evaluated MDPNet on an independent clinical dataset comprising 95 preoperative brain MRI cases provided by the Department of Neurosurgery at Ningxia Medical University General Hospital. The model achieved consistently strong performance on this real-world dataset, further demonstrating its potential and applicability in clinical settings.

In the Kaplan-Meier survival analysis of IDH and ATRX genetic subtypes, although the GBM group did not show a clear survival advantage for mutation-positive patients over wild-type patients, we believe this outcome is primarily due to the extreme class imbalance between these groups. Specifically, in the analysis of IDH subtypes, among the 480 GBM cases included, only 9 patients had an IDH mutation, while the remaining cases were IDH wild-type. Similarly, in the ATRX subtype analysis, only 18 out of 274 GBM cases harbored an ATRX mutation, with the rest classified as ATRX wild-type. Given this imbalance, it would be premature to conclude that IDH and ATRX mutations have no prognostic significance for high-grade glioma patients.

This model can be seamlessly integrated into existing clinical workflows, assisting radiologists in evaluating key genetic subtypes of gliomas based on MRI scans or serving as a decision-support tool for neurosurgeons during diagnosis. Additionally, it holds significant potential for real-time deployment in clinical settings, enabling automated MRI processing for on-the-fly genetic subtype prediction. However, several challenges remain, including the need for standardized imaging protocols across institutions to ensure consistent model performance and the necessity of enhancing model interpretability to build clinicians’ trust in its predictions. By providing a non-invasive, accurate, and rapid method for glioma molecular subtyping, the proposed model could have a profound impact on patient management by improving diagnostic precision, guiding personalized treatment strategies, and reducing the reliance on invasive biopsies. For instance, patients identified as having high-grade gliomas (e.g., IDH wild-type gliomas) could be prioritized for systemic therapy, while those with lower-grade gliomas may benefit from less aggressive treatment approaches. Furthermore, the model’s rapid inference capability allows for timely clinical decision-making. Future research will focus on validating these impacts in real-world clinical environments.

Our study has several limitations. First, our test dataset was obtained from a single medical center, resulting in a relatively small and localized sample. Variations in patient populations and imaging protocols may introduce biases, potentially limiting the generalizability of our findings across diverse clinical settings. Future research should build on the work of Cepeda et al. (37) in multi-center data standardization to minimize imaging discrepancies between different MRI scanners and improve the model’s cross-institutional generalizability. Second, the learning and inference processes of the model remain largely opaque, lacking strong interpretability. Although we have made efforts to enhance model transparency through visualization techniques, further improvements are needed before clinical deployment. Lastly, our study primarily focuses on IDH, 1p/19q, and ATRX, while other glioma-related genetic markers, such as TP53 and MGMT, remain unexplored. Future work will aim to address these challenges by standardizing and validating multi-center data, improving model interpretability, and expanding genetic subtype prediction to include a broader range of glioma-related biomarkers.

## 6 Conclusion

In this study, we introduce MDPNet, a novel network designed to address the challenges of glioma genetic molecular subtyping. This network is capable of performing joint classification of the IDH, 1p/19q, and ATRX genetic molecules. The network is meticulously designed to capture the heterogeneity between T2 and FLAIR modalities, facilitating deep feature extraction and enhancement of glioma imaging characteristics. Additionally, by leveraging inter-gene relationships, MDPNet optimizes the prediction of 1p/19q status while further improving classification performance for IDH and ATRX. To mitigate the adverse effects of data imbalance, we introduce an adaptive weighted loss function, effectively reducing biased predictions during model training. Experimental results demonstrate that the proposed method achieves superior performance on both public and internal datasets, consistently outperforming state-of-the-art models. Furthermore, we conducted a preliminary analysis of the association between ATRX/IDH status and patient survival prognosis using available clinical data. This analysis reinforces the clinical applicability of our approach, highlighting its potential utility in precision oncology. Collectively, these findings suggest that the proposed framework serves as a reliable computer-aided glioma genotyping system for multi-modal MRI-based prediction.

## Data Availability

Publicly available datasets were analyzed in this study. This data can be found here: https://github.com/whz847/MDPNet.
